# Supplementary material for: Transcriptomic Insights into Mechanisms of Early Seed Maturation in the Garden Pea (Pisum sativum L.)
Source: Cells. 2020 Mar 23;9(3):779. doi: 10.3390/cells9030779 (PMC7140803; doi:10.3390/cells9030779)

Zhewan−1,10(4)

Global Summary

%DE = 0.26

genes with fdr < 0.2 = 511 (224 + / 287 −)

genes with fdr < 0.1 = 466 (211 + / 255 −)

genes with fdr < 0.05 = 447 (206 + / 241 −)

genes with fdr < 0.01 = 365 (172 + / 193 −)

genes in genesets = 0

<FC> = 0

<t−score> = 0

<p−value> = 0

<fdr> = 0.74

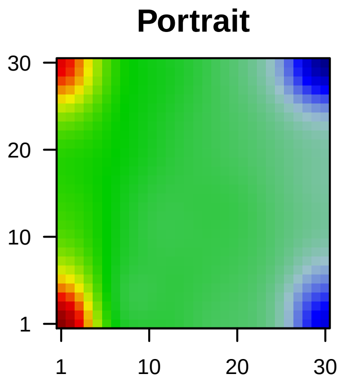

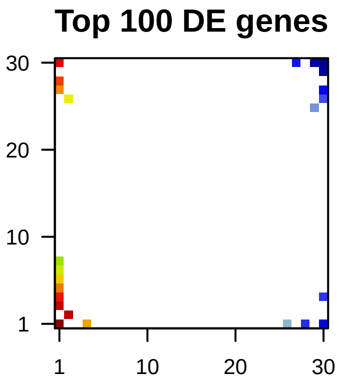

Global Genelist

Rank	ID	log(FC)	p−value	fdr	Metagene	Description
1	TRINITY_DN1	−0.67	2e−16	2e−15	30 x 1	
2	TRINITY_DN1	0.78	2e−16	2e−15	1 x 3	
3	TRINITY_DN2	0.49	2e−16	2e−15	1 x 7	
4	TRINITY_DN9	−0.6	2e−16	2e−15	30 x 30	
5	TRINITY_DN7	−2.46	2e−16	2e−15	30 x 1	
6	TRINITY_DN7	1.21	2e−16	2e−15	30 x 30	
7	TRINITY_DN5	−2.34	2e−16	2e−15	30 x 30	
8	TRINITY_DN5	1.49	2e−16	2e−15	1 x 1	
9	TRINITY_DN5	1.38	2e−16	2e−15	1 x 30	
10	TRINITY_DN7	−0.45	2e−16	2e−15	30 x 30	
11	TRINITY_DN7	1.62	2e−16	2e−15	1 x 1	
12	TRINITY_DN9	−0.49	2e−16	2e−15	30 x 1	
13	TRINITY_DN9	−0.51	2e−16	2e−15	30 x 30	
14	TRINITY_DN9	−0.94	2e−16	2e−15	30 x 30	
15	TRINITY_DN9	−0.52	2e−16	2e−15	30 x 1	
16	TRINITY_DN2	0.59	2e−16	2e−15	1 x 30	
17	TRINITY_DN2	−0.45	2e−16	2e−15	29 x 30	
18	TRINITY_DN2	0.66	2e−16	2e−15	1 x 1	
19	TRINITY_DN2	−0.67	2e−16	2e−15	30 x 1	
20	TRINITY_DN2	−0.5	2e−16	2e−15	30 x 1	

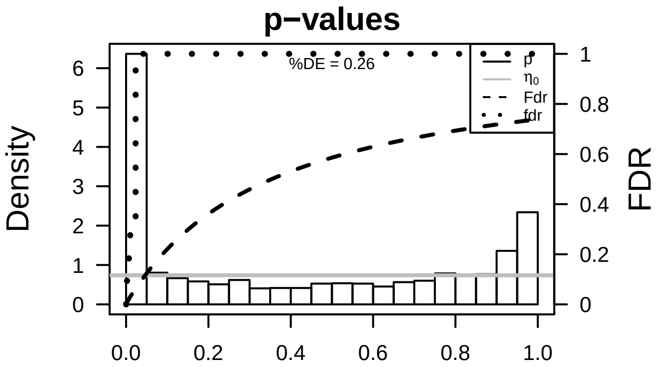

Supplement: Supplementary file 1 [file cells-09-00779-s001.zip › Supplementary materials/FileS4.zip!/FileS3/TFs - Results/Summary Sheets - Samples/Zhewan.1.10.4..pdf]
